# Medaka (*Oryzias latipes*) Embryo as a Model for the Screening of Compounds that Counteract the Damage Induced by Ultraviolet and High-Energy Visible Light

**DOI:** 10.3390/ijms21165769

**Published:** 2020-08-11

**Authors:** Marián Merino, José Luis Mullor, Ana Virginia Sánchez-Sánchez

**Affiliations:** Bionos Biotech SL; Biopolo Hospital La Fe, 46026 Valencia, Spain; mmerino@bionos.es (M.M.); jlmullor@bionos.es (J.L.M.)

**Keywords:** Medaka embryos, reconstructed human epidermis, HaCat cells, UV radiation, HEV light, oxidative stress (ROS), DNA damage, thymine dimers

## Abstract

Continuous overexposure to sunlight increases its harmful effects on the skin. For this reason, there is a growing need to characterize economic models more representative of the negative effects and counteracting responses that irradiation causes on human skin. These models will serve for the screening of protective compounds against damage caused by ultraviolet (UV) and high energy visible light (HEV). Therefore, two common in vitro models employed for sunlight irradiation studies, namely human keratinocyte HaCat culture and reconstructed human epidermis (RHE), were compared with the medaka fish embryo model, traditionally used in other scientific disciplines. Using suberythemal doses of UVA and HEV to determine the level of Reactive Oxygen Species (ROS) generation and thymine dimers formed by UVB, we show that medaka embryo responds with a lower damage level, more comparable to human skin, than the other two models, probably due to the protective mechanisms that work in a complete organism. In the same way, the protective effects of antioxidant compounds have the greatest effect on medaka embryos. Taken together, these findings suggest that medaka embryos would be a good alternative in vitro model for sunlight effect studies, and for the screening of molecules with counteracting capacity against the damage caused by UV and HEV.

## 1. Introduction

Ultraviolet (UV) radiation from the sun is a natural source of energy with proven benefits for humans, such as the production of Vitamin D in the skin triggered by UVB light (290–320 nm), which is important for normal bone formation. UV radiation is also used as a therapy for skin diseases like atopic dermatitis or psoriasis [1]. However, UV radiation can be very harmful causing serious skin problems, such as skin burns, oxidative stress imbalance, inflammation, immune alterations, or DNA damage [2]. Moreover, injury caused by UV exposure is cumulative, leading to premature skin aging and, even in the worst case, skin cancer like melanoma [3].

One of the main contributors to skin photoaging is the formation of ROS that induce oxidative stress. In the skin, ROS generation is due by both intrinsic and extrinsic factors, being UV radiation one of the most important extrinsic activators of endogenous photosensitizers to generate ROS [4]. Nevertheless, UV radiation contains less of the 10% of solar radiation, whereas visible light (400–700 nm) comprises around 43%, and has been reported also as an important ROS producer in the skin, contributing to signs of premature photoaging due to the oxidative stress imbalance [5]. Additionally, new man-made sources of visible light are widely present in our lives due to technological evolution, such as screens of electronic devices or LED lighting, that also contribute to skin damage [6].

Skin aging is also associated with DNA damage, which can lead to skin cancer. Long-term overexposure to sunlight has direct and indirect toxicity effects on the DNA [7,8]. The indirect DNA damage is also linked to ROS generation, as ROS can damage many cellular molecules such as proteins, lipids, and DNA. When UVA photons reach the skin cells, they transfer electrons from endogenous photosensitizers, creating hydroxyl radicals, singlet oxygen anion, and hydrogen peroxide [9]. On the other hand, UVB is responsible for the direct DNA toxicity, due to the absorption of UVB photons by the DNA bases, mainly pyrimidine components that includes cytosine and thymine. The consequence of this is the structural reorganization of nucleotides that causes double-strand aberrations [10]. At this moment, cells initiate the DNA repair mechanism and immediately stop cell division to prevent additional DNA damage. In mammals, this mechanism involves the nucleotide excision repair pathway [11].

One of the most widespread in vitro models used to study the effects of solar radiation on the skin is the two-dimensional (2D) culture of human keratinocyte or fibroblasts cells [12]. These models have the advantage of being low cost and timesaving. Nevertheless, by presenting only one cell type in monolayer culture, they diverge from the real structure of the skin and its interactions between the different cells, intercellular matrix, and other tissues [13]. Three-dimensional (3D) cultures of skin equivalents are a well-established alternative to monolayer cell cultures. These 3D cultures can be obtained in-house, and they are also commercially available [14,15], but lack tissue interactions and are expensive to obtain. In the same way, porcine skin has become an important model of human skin due to the functional and anatomical resemblance. However, its availability and handling can be complicated to manage for routine use [16,17].

In this situation arises the need to develop and validate new alternative methodologies that could be more representative of the complex interactions that occur in the human body. For this reason, the use of fish embryos, like Zebrafish or Medaka fish, as an alternative in vitro model provides many advantages [18,19]. This model has been extensively used in several research disciplines such as development biology [20], human disease model [21,22,23], and even screening for antioxidant molecules [24] or functional food [25]. Medaka fish embryos have a great number of advantages to be used as a screening platform for antioxidant and sunlight protective products, such as their economic maintenance and experimentation, their small size and transparent chorion that allow researchers to observe them using a simple loupe, the availability of a high number of daily embryos, and the presence of all cell types, tissue and organs interactions [26]. Additionally, teleost fishes have three layers in their skin, which include stratified epithelial cells in the epidermis, fibroblast in the dermis, and other skin cells such as melanophores (similar to melanocytes in mammals), mast cells, and Merkel cells. Epidermis and dermis are present from early stage of embryo development in teleost fish [27]. Moreover, many essential functions are shared between human and medaka fish skin, for example, innate immune mechanisms for dealing with pathogens, or eumelanin synthesis in melanophores that starts to be visible in medaka embryos at stage 28 [19,28,29]. However, other functions in teleost fish are not present in mammals as, for example, photoenzymatic repair, which reverses the formation of UVB or UVC-induced adjacent cyclopyrimidine dimers [30].

In this paper, we compare the suitability of medaka fish embryos for studying the harmful effects of exposure to sunlight (focusing on ROS production and DNA damage), comparing with monolayer cell cultures of immortalized keratinocytes (HaCat cells) and 3D culture of the reconstructed human epidermis (RHE) formed by a stratified epithelium of keratinocytes. These three models generated ROS as a reaction of UVA and visible light exposure. Furthermore, thymine dimers have been quantified to determine the level of DNA damage produced by UVB irradiation also in the three models. Additionally, we have evaluated the effect of three antioxidant compounds: retinoic acid (Vit A), ascorbic acid (Vit C) and resveratrol [31,32,33], counteracting levels of ROS generated by UVA and HEV light in medaka embryos and HaCat cell culture, as these two models have the advantage of a similar low economic cost, making them suitable for use as compound screening platforms.

## 2. Results

### 2.1. Evaluation of DNA Damage Generated by UVB in HaCat Culture, RHE and Medaka Embryos

**Figure 1:**
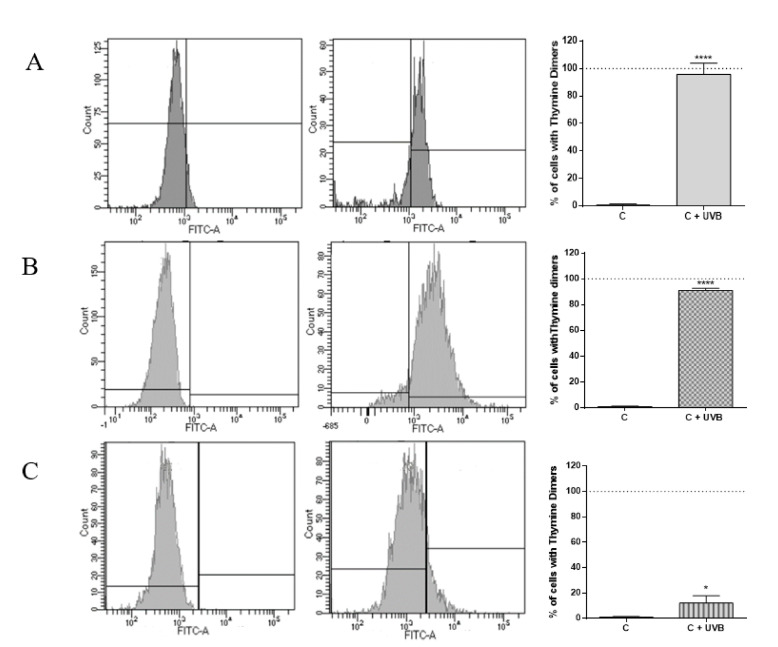
Histograms of one sample of each group included in the experiment, non-irradiated (left histograms) or irradiated with 0.04 J/cm^2^ UVB (right histograms), and Barr graph showing the percentage of thymine dimers generated in each condition for HaCat culture cells (**A**), RHE (**B**) and medaka embryos (**C**). C—control samples and C+UVB—control samples irradiated with UVB. * *p* < 0.05; **** *p* < 0.0001.

To determine the effect of UVB irradiation on generating DNA damage by measuring the thymine dimers accumulation, HaCat cells, RHE, and fish embryos were exposed to a total dose of 0.04 J/cm^2^. This dose was chosen as it was the minimum dose necessary to observe a significant increase in thymine dimers in medaka. Interestingly, the same dose caused at least 20 cyclobutene dimers/10^6^ nucleotides in the skin of healthy human volunteers [34]. Results indicated that UVB irradiation significantly induced DNA lesions in the form of thymine dimers compared to non-irradiated controls. Applying the same UVB dose to each model, it can be appreciated that HaCat cells recorded the highest significant increase in the percentage of thymine dimers compared to non-irradiated cells (94.7 ± 8.1%). This could be explained considering that this culture is composed of only one cell type growing in monolayer, and therefore this model lacks all the interactions that take place in the skin. In addition, cells growing in a monolayer are more exposed to radiation damage than if they were forming the entire structure of a tissue. On the other hand, RHE also showed a high accumulation of thymine dimers (90.3 ± 1.5%) after UVB exposure. Nevertheless, this value was lower than for the 2D culture, confirming that as the complexity of the culture increases, it mimics more adequately in vivo conditions. Finally, in the case of medaka fish embryos, the level of thymine dimers significantly increased in 11.1 ± 3.3%. This value is below those obtained in the other models, in which almost all cells have thymine dimers, probably because in medaka embryos, all the interactions between the different components of a whole organism take place and DNA protection mechanisms can work appropriately (Figure 1).

### 2.2. Evaluation of ROS Generation by UVA and Visible Light in HaCat Culture, RHE and Medaka Embryos

To establish the levels of ROS produced by high energy visible light (HEV, 400–460 nm) and UVA radiation, HaCat cells, RHE, and fish embryos were irradiated with a suberythemal dose of 10 J/cm^2^ for UVA, or 50 J/cm^2^ for HEV. The suberythemal UVA dose was selected based on the minimal erythematous dose that produces inflammation in the most sensitive human skin phototype (phototype I, bright white skin color), which is 15 J/cm^2^ [35]. On the other hand, Vandersee et al. [36] showed that, in the skin of human volunteers irradiated with HEV, 50 J/cm^2^ was the dose needed for significant degradation of carotenoids that indirectly showed the amount of ROS.

**Figure 2:**
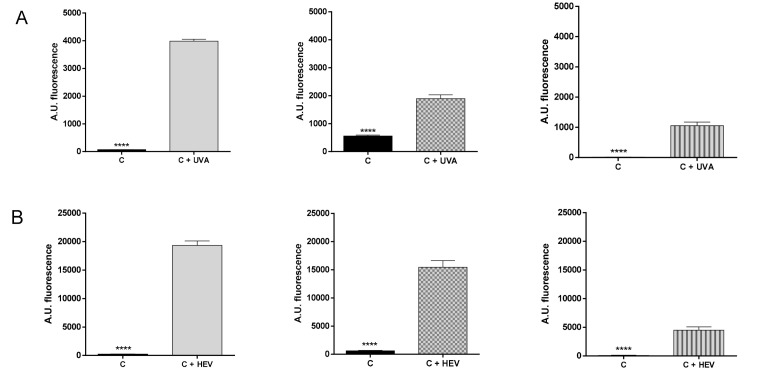
Barr graph showing ROS levels generated for HaCat culture cells (grey bar), RHE (grey bar with squares), and medaka embryos (grey bar with lines) after UVA radiation (**A**) and HEV radiation (**B**). C—control samples, C+UVA—control samples irradiated with UVA and C+HEV—control samples irradiated with HEV. **** *p* < 0.0001.

After samples irradiation with UVA or HEV, ROS levels were measured to determine the oxidative damage generated in each model (Figure 2). For UVA irradiation (Figure 2A), HaCat cells had the highest ROS accumulation (3919 ± 66 Arbitrary Units (AU)) in comparison with the non-irradiated cells. As expected, a one-cell culture is more exposed to irradiation than a more complex system formed by different types of cells and layers, and where the different mechanisms to solve an imbalance of oxidative species could act and repair the damage generated. Regarding RHE, ROS levels significantly increased by 1333 ± 140 AU, but this increase was lower than in HaCat cell monolayer culture, probably because the RHE model constitutes a more complete simulation of human skin composed by several layers of keratinocyte cells that can interact and react faster against damage. Last, medaka embryos had lower ROS accumulation than the other two models, having a difference with non-irradiated embryos of 1041 ± 121 AU.

On the other hand, when HaCat cells, RHE, and fish embryos were exposed to HEV radiation, it could be observed in the same pattern (Figure 2B). The monolayer culture of HaCat showed the highest ROS accumulation, having a difference of 19081.0 ± 784.1 AU in comparison to non-irradiated cells. For RHE, the damage generated was reduced by 25% in comparison with the 2D culture and the difference with RHE non-irradiated was 22547 ± 3928 AU. In the case of medaka embryos, the total ROS levels accumulated were 75% less than in HaCat, showing an increase of 4418.0 ± 561.6 AU with embryos not exposed to HEV. Taking together, these results highlight the differences that exist between in vitro models, and the importance of using a model that mimics the effect of radiation in the human skin. Regarding this fact, the medaka embryos seem to show a more moderate ROS level provoked by UVA and HEV than the other models, more in line with the damage that the tested UVA and HEV doses cause in human skin [36,37].

### 2.3. Testing of Antioxidant Compounds after UVA and HEV Radiation in HaCat Culture and Medaka Embryos

**Figure 3:**
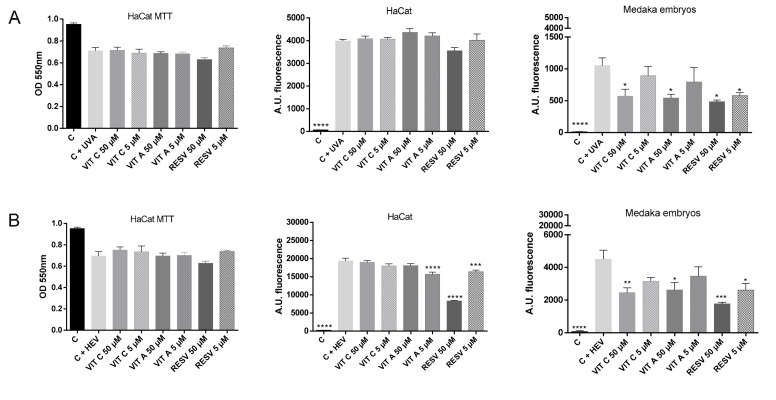
Barr graph showing ROS levels generated for HaCat culture cells and medaka embryos treated with three antioxidant compounds, after UVA radiation (**A**) and HEV radiation (**B**). VIT C—vitamin C, VIT A—vitamin A and RESV—resveratrol. * *p* < 0.05; ** *p* < 0.01; *** *p* < 0.001; **** *p* < 0.0001.

Considering the previous results, medaka embryos could be an alternative economic platform for screening of compounds capable of counteracting the harmful effects of UV radiation and HEV light. To evaluate this possibility, we compared the effect of three well-known antioxidant compounds in medaka embryos and HaCat culture, as this last model is a typical platform used for compound screening [38,39,40]. The selected antioxidant compounds were vitamin C (ascorbic acid), vitamin A (retinoic acid), and resveratrol [41,42,43,44,45]. For each compound, two non-toxic concentrations (5 and 50 μM) were chosen after performing a viability assay in medaka embryos and MTT assay in HaCat cells. Compounds were applied for 24 h for HaCat cells and 2 h for medaka embryos. After the incubation period with each compound, medaka embryos and HaCat cells were irradiated with UVA or HEV, and ROS levels were measured. As shown in Figure 3, cell viability was reduced by both types of irradiation, with the compounds being unable to counteract this effect in cell viability. On the other hand, ROS highly increased in both HaCat culture and medaka embryo models irradiated with UVA and HEV.

Focusing on UVA results, compounds did not reduce levels of ROS in HaCat cells (Figure 3A). Interestingly, in medaka embryos, it could be observed a protective effect of antioxidant compounds. Moreover, ROS levels were decreased in a dose-dependent manner, being the high concentration (50 μM) the one that significantly decreased the accumulation of ROS in vitamin C and Vitamin A. In the case of resveratrol, both concentrations (50 μM and 5 μM) presented a protective effect against the oxidative damage generated by UVA radiation, indicating that resveratrol antioxidant ability is higher than Vitamins A and C in medaka embryos.

Regarding HEV radiation (Figure 3B), a similar effect was observed. ROS levels highly increased after radiation, meaning that this dose of HEV was adequate for generating oxidative damage. For HaCat monolayer culture, vitamin C did not show any protective effect and vitamin A decreased ROS levels only at 5 μM concentration. However, resveratrol produced a high antioxidant power, reducing ROS accumulation at both concentrations (50 μM and 5 μM). On the other hand, medaka embryos provided a better discrimination method for antioxidant ability as ROS levels were reduced depending on concentration, showing that, as occurred with UVA radiation, resveratrol was the only compound that could decrease ROS levels at both tested concentrations.

These results demonstrate that medaka embryos could constitute a good model for the evaluation of protective effects against radiation by reducing ROS, as it is a more complete model for simulating in vivo reaction and response to repair the damage produced by radiation.

## 3. Discussion

Overexposure to sunlight causes significant damage to the skin, such as oxidative stress imbalances, inflammation, immune alterations, or DNA damage. These negative impacts are cumulative and repeated exposures lead to premature skin aging and the appearance of skin cancer [3]. For these reasons, it is necessary to develop economic platforms that respond similarly to human skin when exposed to sunlight, and that allows for the screening of compounds capable of bearing the different effects suffered by human skin exposed to sunlight. In this paper, we compare two habitual in vitro models used in human skin research, HaCat culture cells and RHE, with Medaka embryo, which is also considered as an in vitro model [19].

This complexity in structure and cell type content does not exist in 2D culture models or RHE, where only one type of cell is included, lacking cell and tissue interactions. Probably due to these facts, the results obtained using the same doses in the three models showed less impact of UVA and HEV on ROS formation and thymine dimer accumulation generated by UVB in medaka embryo than in HaCat or RHE cultures.

Sunlight penetrates the skin in a wavelength-dependent manner, and therefore, UVB is mainly absorbed by the epidermis, whereas UVA and HEV can penetrate dermis [46]. The skin structure of medaka embryos, like human skin, implies that the radiation effects can occur in the same layers in both, which differs from monolayer and RHE cultures, where fibroblasts and dermis are not present. UVA and HEV produce ROS in epidermis and dermis of medaka embryos and humans. In human skin, ROS accumulation trigger a protein activation cascade that finally induces the expression of MMP1, which is responsible for increased extracellular protein degradation, and the inhibition of *TGF-β* which is responsible for collagen production [4,47]. This mechanism of extracellular matrix remodeling was similarly described in medaka ovary for follicle rupture during ovulation, where MMP proteins action is necessary for degrading collagen type 1 [48]. It would be very interesting to study whether this pathway is also acting in the medaka embryo skin after UV exposure.

ROS provokes proteins carbonylation, lipid peroxidation, and DNA damage that could lead to cell death [4]. Our results showed than approximately 20–25% of HaCat cells irradiated with UVA or HEV died during irradiation, however, all medaka embryos survived to the same doses of HEV and UVA irradiation, probably because generated ROS levels were lower in medaka embryos than in HaCat cells. One explanation for the difference in lethality and ROS generation between the three compared models could be that medaka embryo skin have melanophores (equivalents to the human melanocytes), which are not present in HaCat or RHE cultures. Melanocytes synthesize melanin, which has antioxidant properties, works as an absorbent filter that reduces the penetration of UV in the skin, and functions as a free radical scavenger [49]. In medaka, there are more than 40 pigmentation mutations available, some of them without melanin, or with only a low level [50] than could be used for studying the role of melanin in medaka embryo skin against sunlight exposure, and compared with those of melanin in human skin.

In a different way, UVB generates direct DNA damage in epidermal cells, mainly in the form of pyrimidine dimers. In mammals, nucleotide excision repair is activated in the response of this UV insult [51]. This mechanism of DNA repair is present in teleost fish, which like in mammal cells, UVB treatment promotes H2AX phosphorylation and a p53-dependent response [52]. A similar dose of UV that we used for generating thymine dimers in medaka embryos was used by Bykov et al. [34] to obtain a quantifiable level of cyclobutene dimers in human volunteers. However, levels were higher in HaCat cells and RHE, in which almost all cells had thymine dimers. Taking all the results together, medaka embryos could be a good model to study the impact of solar radiation on the skin and the mechanisms that the skin activates to counteract them since it is a simple to use and economic model that shares the complex structure of the skin with humans.

Medaka embryos are used for toxicity assays, screening of antioxidant molecules [24], or functional food [25]. Therefore, we wanted to compare the two more economic models as platforms for protective molecules against sunlight irradiation. For this reason, HaCat cells and medaka embryos were treated with the same concentrations of three well-characterized antioxidants [41,42,43,44,45], before UVA and HEV exposure. In both models, irradiations generated ROS increase, being higher in HaCat cells than in medaka embryos. Interestingly, the effects of antioxidants were more pronounced in medaka than in HaCat. In fact, vitamin C did not show any protective effect in HaCat, despite it works as an antioxidant in the skin of human volunteers [53,54]. Interestingly, vitamin C reduced the level of ROS produced by UVA and HEV in medaka embryos.

In conclusion, medaka embryos could be used as an economic in vitro model to study how the different damages generated by sunlight develop, and how the skin responds to these damages. In addition, it would also be a good platform for screening protective molecules against damage caused by sunlight.

## 4. Materials and Methods

### 4.1. Cell Culture

Human keratinocyte cell line (HaCat) were cultured in Gibco DMEM medium, low glucose (Fisher scientific, Madrid, Spain) supplemented with l-Glutamine (Sigma Aldrich, Darmstadt, Germany), penicillin-streptomycin (Gibco, Fisher Scientific, Madrid, Spain) and fetal bovine serum (Gibco, Fisher Scientific, Madrid, Spain). Cultures were maintained at 37 °C with a 5% CO_2_ humidified atmosphere. To examine antioxidant effects, three compounds at different concentrations were selected based on the bibliography. The chosen compounds were Ascorbic acid (Vit C), retinoic acid (Vit A), and resveratrol (Sigma Aldrich, Darmstadt, Germany). Prior to UV radiation, cells were incubated 24 h under standard conditions in medium containing 50 μM or 5 μM of each compound. Vit A was diluted in a non-toxic concentration of methanol and Resveratrol in DMSO. The concentrations used in these assays were not cytotoxic previously determined by the MTT assay.

### 4.2. Reconstructed Human Epidermis EpiDerm^TM^ (MatTek)

RHE EpiDerm^TM^ 3D skin model (size 0.33 cm^2^) was acclimatized for 24 h immediately after reception. After this incubation, RHE samples were irradiated with UVB to induce DNA damage and thymine dimers accumulation or with UVA or HEV to generate ROS. After each irradiation, RHE samples were processed as described below.

### 4.3. Fish Embryos

Adult medaka (*Oryzias latipes*) CAB strain animals were maintained in recirculating water aquaria on a 14 h light/10 h dark daily cycle at 28 °C. Fish embryos were collected by natural spawning in Yamamoto buffer (NaCl, CaCl_2_, NaHCO_3_ from Sigma-Aldrich, KCl from USB). They were raised at 25 °C and were staged as described [29]. The same compounds chosen for cell assays were used in this experiment. Embryos at stage 32 were treated with 5000–500—50–5 μM of Vit C, Vit A, or resveratrol (Sigma Aldrich, Darmstadt, Germany). Five replicates of every condition were used and untreated embryos (control) were maintained in Yamamoto buffer. Every hour, embryos were observed to determine their survival or death. Then, 50 and 5 μM were chosen for the irradiation experiments as these concentrations were non-toxic in fish embryos. Prior to UV radiation, fish embryos were incubated 2 h in Yamamoto containing 50 μM or 5 μM of each compound.

### 4.4. Flow Cytometric Analysis of Thymine Dimers (DNA Damage)

To evaluate the capacity of UVB radiation to induce the development of DNA lesions, thymine dimers formation was quantified by flow cytometry. After UVB radiation with a total dose of 0.25 J/cm^2^, HaCat cells, RHE, or fish embryos were incubated 10 min. After that, HaCat cells, RHE, and embryo cells were disaggregated with trypsin at 37 °C and fixed with 4% paraformaldehyde (PFA) (Electron Microscopy Sciences, Madrid, Spain). Following, they were incubated with primary antibody mouse monoclonal anti-Thymine Dimer (H3) (Abcam, Cambridge, UK) and stained with secondary antibody Alexa 488 labeled goat anti-mouse IgG (Invitrogen, Fisher Scientific, Madrid, Spain). Finally, samples were analyzed by BD Flow Cytometer (BD Biosciences, San Jose, CA, USA).

### 4.5. MTT Assay

To evaluate cell viability, 3-(4,5-dimethylthiazol-2-yl)-2,5-diphenyltetrazolium bromide (Sigma Aldrich, Darmstadt, Germany) was used. HaCat cells treated with the different compounds and UVA, UVB or HEV irradiated, were incubated with the MTT reagent for 3 h. After the incubation period, dimethyl sulfoxide (Sigma Aldrich, Darmstadt, Germany) was used to dissolve the formazan and the color intensity was measured at 550 nm in a spectrophotometer Halo LED 96 (Dynamica Scientific, Livingston, UK).

### 4.6. UV Irradiation

The light source was supplied by a Luzchem Exposure Panel EXPO-01 (Luzchem, Ontario, Canada) that hold five 8-Watt fluorescent lamps: UVA lamps are centred at approximately 300 nm with a radiation peak at 368 nm, UVB lamps are centred at approximately 350 nm with a radiation peak at 306 nm and HEV lamps were centred at 445–465 nm (Fluorimport, Peschiera Borromeo, Italy). The dosage used for each radiation was 10 J/cm^2^ for UVA, 0.04 J/cm^2^ for UVB, and 50 J/cm^2^ for HEV. HaCat cells were irradiated in culture medium and the irradiation panel was located at the bottom of the plate. On the other hand, RHE and fish embryos were irradiated from the top with a distance from the irradiator panel of 10 cm. Parallel HaCat cells, RHE, or fish embryos were maintained without irradiation (non-irradiated controls).

### 4.7. ROS Detection

For the detection of intracellular ROS generated due to the radiation, the Fluorometric Intracellular Ros kit (Sigma Aldrich, Darmstadt, Germany) was used following the manufacturer’s instructions. HaCat cells, RHE, or medaka fish embryos were incubated with ROS reagent, and immediately after the addition of it, they were irradiated and fluorescence was measured in a GloMax Microplate Reader (Promega, Madrid, Spain).

### 4.8. Statistical Analysis

GraphPad Prism software, version 6 (GraphPad, San Diego, CA, USA) was used to perform the statistical analysis. Data are represented as mean ± SEM and the test applied for the analysis was the ordinary one-way ANOVA test with Dunnet post-Hoc and unpaired Student’s *t*-test. Statistical significance was set at *p* < 0.05, 95% of confidence.

## Abbreviations

AU	Arbitrary units
HEV	High Energy Visible light
RHE	Reconstructed human epidermis
ROS	Reactive Oxygen Species
UV	Ultraviolet
